# Improvement of the SOD activity of the Cu^2+^ complexes by hybridization with lysozyme and its hydrogen bond effect on the activity enhancement

**DOI:** 10.3389/fchem.2023.1330833

**Published:** 2024-01-18

**Authors:** Daisuke Nakane, Yukihito Akiyama, Soma Suzuki, Ryotaro Miyazaki, Takashiro Akitsu

**Affiliations:** Department of Chemistry, Faculty of Science, Tokyo University of Science, Tokyo, Japan

**Keywords:** Cu^2+^ complex, SOD activity, hybrid protein, lysozyme, hydrogen bond

## Abstract

We prepared *L*-amino acids (*L*-valine and *L*-serine, respectively) based on the Schiff base Cu^2+^ complexes CuSV and CuSS in the absence/presence of hydroxyl groups and their imidazole-bound compounds CuSV-Imi and CuSS-Imi to reveal the effects of hydroxyl groups on SOD activity. The structural and spectroscopic features of the Cu^2+^ complexes were evaluated using X-ray crystallography, UV-vis spectroscopy, and EPR spectroscopy. The spectroscopic behavior upon addition of lysozyme indicated that both CuSV and CuSS were coordinated by the imidazole group of His15 in lysozyme at their equatorial position, leading to the formation of hybrid proteins with lysozyme. CuSS-Imi showed a higher SOD activity than CuSV-Imi, indicating that the hydroxyl group of CuSS-Imi played an important role in the disproportionation of O_2_^−^ ion. Hybridization of the Cu^2+^ complexes CuSV and CuSS with lysozyme resulted in higher SOD activity than that of CuSV-Imi and CuSS-Imi. The improvements in SOD activity suggest that there are cooperative effects between Cu^2+^ complexes and lysozyme.

## 1 Introduction

Reactive oxygen species (ROS), such as hydroxyl radicals (^•^OH), singlet oxygen (^1^O_2_), hydrogen peroxide (H_2_O_2_), and superoxide (O_2_^−^) are unavoidable byproducts of respiration in aerobic organisms. These ROS cause serious oxidative damage to biomolecules such as lipids, proteins, and nucleic acids. Among these, O_2_^−^ is generated by the one-electron reduction of dioxygen (O_2_) upon enzymatic oxidation and oxygen delivery (Pryor, 1986). In addition to damaging biomolecules, O_2_^−^ also induces the generation of other ROS, such as ^•^OH and H_2_O_2_, under protic conditions (Hayyan et al., 2016). To remove O_2_^−^ from their bodies, organisms have superoxide dismutase (SOD), which catalyzes the disproportionation of O_2_^−^ to H_2_O_2_ and O_2_, as shown below.

$$2O_{2}^{-}+2H^{+}\;\to \;H_{2}O_{2}+O_{2}$$

As SODs play an important role in protecting biomolecules from oxidative damage, animals with higher SOD activity generally have a longer life expectancy (Tolmasoff et al., 1980).

SODs contain metal ion(s) in their active centers and catalyze the disproportionation of O_2_^−^ as shown below.

$$M^{n+}+O_{2}^{-}+2H^{+}\;\to \;M^{\left(n+1\right)+}+H_{2}O_{2}$$

$$M^{\left(n+1\right)+}+O_{2}^{-}\;\to \;M^{n+}+O_{2}$$

At present, SODs typically contain Mn, Fe, Ni, Cu, and Zn as their central metals. SODs can be classified as MnSOD (McCord and Fridovich, 1969; Abreu et al., 2005; Kanematsu and Asada, 1979; Fréalle et al., 2005; del Rio et al., 2003; Sheng et al., 2014), FeSOD (Yost and Fridovich, 1973; Hatchikian and Henry, 1977; Opperdoes et al., 1977; Kirby et al., 1981; Pilon et al., 2011), NiSOD (Youn et al., 1996a; 1996b; Palenik et al., 2003; Barondeau et al., 2004; Wuerges et al., 2004), and CuZnSOD (Mann and Keilin, 1938; Goscin and Fridovich, 1972; Alscher et al., 2022), respectively. In this study, we focused on CuZnSOD, which has a dimetallic active center composed of Cu^2+^ and Zn^2+^ ions. While Zn^2+^ ions fix the secondary coordination sphere of the active center of CuZnSOD, Cu^2+^ ions catalyze the disproportionation of O_2_^−^ ions, as shown below (Hart et al., 1999).

$${\text{Cu}}^{2+}+O_{2}^{-}\;\to \;{\text{Cu}}^{+}+O_{2}$$

$${\text{Cu}}^{+}+O_{2}^{-}+2{Η}^{+}\;\to \;{\text{Cu}}^{2+}+{Η}_{2}{Ο}_{2}$$

However, to apply native CuZnSOD as an O_2_^−^ removing reagent, problems such as its poor stability and high cost must be addressed (O’Connor et al., 2012). To address these problems, hybridization of proteins and SOD-active metal complexes with small molecular weights has gained significant attention. In practice, ROS-removing ability is reported in Cu^2+^-bound albumin (Kato et al., 2014).

In this study, we focused on lysozyme because of its stability under ambient conditions. In addition, the His15 side chain of lysozyme is known as the binding site of metal ions such as Mn^+^ (Razavet et al., 2007), Ag^+^ (Panzner et al., 2011), Au^+^ (Ferraro et al., 2019), and Pt^2+^ (Casini et al., 2007).

**FIGURE 1:**
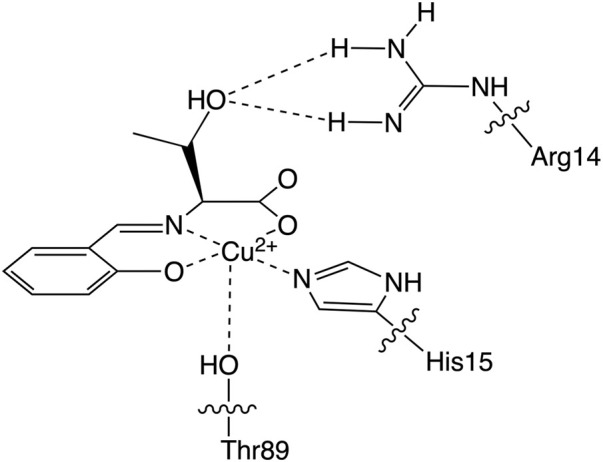
The Cu^2+^ center structure of the hybrid protein composed of the Cu^2+^ complex (CuST) and lysozyme.

Based on these lysozyme properties, we have reported a hybrid protein composed of an SOD-active Cu^2+^ complex coordinated by a threonine derivative (CuST) and lysozyme (CuST@lysozyme), as shown in Figure 1 (Furuya et al., 2023). We determined its crystal structure, spectroscopic and electrochemical properties, and SOD activity. According to the crystallographic analysis, the Cu^2+^ center of CuST@lysozyme was coordinated by the imidazole group of His15 and the hydroxyl group of Thr89 residues of the lysozyme at the equatorial and apical positions, respectively. In addition, the hydroxyl group of CuST formed hydrogen bonds with the side chain of Arg14 of the lysozyme. The hybrid lysozyme exhibited SOD activity comparable to that of CuST.

**FIGURE 2:**
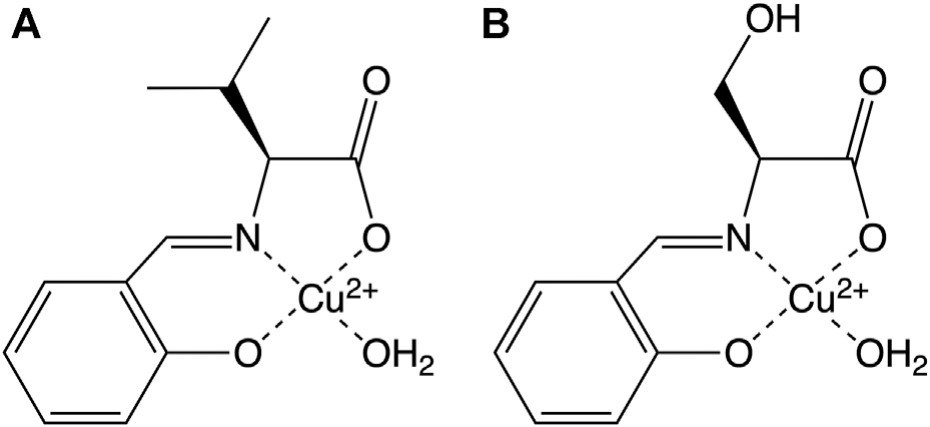
Schematic structure of **(A)** CuSV and **(B)** CuSS prepared in this study.

Since we speculated that the hydrogen bonds formed between the hydroxyl group of the Cu^2+^ complex and the Arg14 residue of lysozyme play an important role in the SOD activity, we prepared two Cu^2+^ complexes in the absence/presence of the hydroxyl group (CuSV and CuSS, respectively), as shown in Figure 2 to evaluate the hydrogen bond effects on their SOD activity. We revealed their crystal structure and spectroscopic and electrochemical properties and investigated their hybridization with lysozyme. In addition, we evaluated the O_2_^−^ disproportionation activity of Cu^2+^ complex-bound hybrid lysozymes and discussed the effects of hydrogen bonds on SOD activity.

## 2 Materials and methods

### 2.1 Materials

Salicylaldehyde was purchased from Tokyo Chemical Industry Co., Ltd. (Tokyo, Japan), and the solvents were purchased from Kanto Chemical Co., Inc. (Tokyo, Japan). Other reagents were purchased from FUJIFILM Wako Pure Chemical Corporation (Osaka, Japan). All reagents were of the highest commercial grade and used without further purification.

### 2.2 Preparation of Cu^2+^ complexes

#### 2.2.1 Preparation of CuSV

*L*-valine (118.0 mg, 1.01 mmol) and salicylaldehyde (121.5 mg, 1.00 mmol) were dissolved in 20 mL of methanol. The solution was subjected to microwave irradiation (Otani et al., 2021) at 358 K for 5 min to yield a yellow ligand solution. Copper (II) acetate monohydrate (200.9 mg, 1.01 mmol) was added to the solution and additionally treated with microwave irradiation at 358 K for 5 min to yield a green solution. The solution was placed under ambient conditions for 3–5 days to obtain green crystals suitable for X-ray analysis (yield: 135 mg, 43.8%).

The elemental analysis results calculated for C_12_H_15_NO_4_Cu are as follows: C: 47.92%, H: 5.03%, N: 4.66%, found, C: 47.78%, H: 5.12%, N: 4.61%. IR:1,468 cm^−1^ (*ν*_sym_ (C=O)), 1,600 cm^−1^ (*ν*_*a*s_ (C=O)), 1,639 cm^−1^ (*ν* (C=N)), 3,232 cm^−1^ (*ν* (O−H)).

#### 2.2.2 Preparation of CuSV-Imi

*L*-valine (117.1 mg, 1.00 mmol) and salicylaldehyde (122.2 mg, 1.00 mmol) were dissolved in 20 mL of methanol. The solution was subjected to microwave irradiation at 358 K for 5 min to yield a yellow ligand solution. Copper (II) acetate monohydrate (200.0 mg, 1.00 mmol) was added to the solution and additionally treated with microwave irradiation at 358 K for 5 min to yield a green solution. Imidazole (71.5 mg, 1.05 mmol) was added to the green solution, which was then microwave-irradiated at 358 K for 5 min to yield a dark green solution. The solution was placed under ambient conditions for 3–5 days, and dark-green crystals suitable for X-ray analysis were obtained (yield: 180.5 mg, 51.6%).

The elemental analysis results calculated for C_15_H_17_N_3_O_3_Cu are as follows: C: 51.35%, H: 4.88%, N: 11.98%, found, C: 51.27%, H: 4.92%, N: 12.03%. IR:1,465 cm^−1^ (*ν*_sym_ (C=O)), 1,586 cm^−1^ (*ν*_*a*s_ (C=O)), 1,632 cm^−1^ (*ν* (C=N)).

#### 2.2.3 Preparation of CuSS

*L*-serine (110.0 mg, 1.05 mmol) and salicylaldehyde (129.2 mg, 1.06 mmol) were dissolved in 20 mL of methanol. The solution was subjected to microwave irradiation at 358 K for 5 min to yield a yellow ligand solution. Copper (II) acetate monohydrate (203.0 mg, 1.02 mmol) was added to the solution and additionally treated with microwave irradiation at 358 K for 5 min to yield a green solution. The solution was placed under ambient conditions for 3–5 days to obtain green crystals (yield: 135 mg, 43.8%).

The elemental analysis results calculated for C_10_H_11_NO_5_Cu·H_2_O are as follows: C: 39.15%, H: 4.27%, N: 4.57%, found, C: 38.98%, H: 4.25%, N: 4.50%. IR:1,465 cm^−1^ (*ν*_sym_ (C=O)), 1,602 cm^−1^ (*ν*_*a*s_ (C=O)), 1,628 cm^−1^ (*ν* (C=N)), 3,132, 3,236, 3,236 cm^−1^ (*ν* (O−H)).

#### 2.2.4 Preparation of CuSS-Imi

*L*-serine (110.0 mg, 1.05 mmol) and salicylaldehyde (126.6 mg, 1.04 mmol) were dissolved in 20 mL of methanol. The solution was subjected to microwave irradiation at 358 K for 5 min to yield a yellow ligand solution. Copper (II) acetate monohydrate (201.6 mg, 1.01 mmol) was added to the solution and additionally treated with microwave irradiation at 358 K for 5 min to yield a green solution. Imidazole (68.9 mg, 1.01 mmol) was added to the green solution, which was then microwave-irradiated at 358 K for 5 min to yield a dark green solution. The solution was placed under ambient conditions for 3–5 days to obtain dark green crystals (yield: 150.7 mg, 44.6%).

The elemental analysis results calculated for C_13_H_13_N_3_O_4_Cu are as follows: C: 46.09%, H: 3.87%, N: 12.40%, found, C: 45.74%, H: 3.94%, N: 12.34%. IR: 1,462 cm^−1^ (*ν*_sym_ (C=O)), 1,600 cm^−1^ (*ν*_*a*s_ (C=O)), and 1,626 cm^−1^ (*ν* (C=N)), 3,134 cm^−1^ (*ν* (O−H)).

### 2.3 Physical measurement

Microwave synthesis was performed using a Biotage initiator+. Elemental analyses (C, H, and N) were performed using a Vario EL cube analyzer at the Nagoya Institute of Technology. IR spectra were recorded on a JASCO FT-IR 4200 spectrophotometer in the range of 4,000–400 cm^−1^ at 298 K. UV–vis spectra were measured using a JASCO V-570 spectrophotometer in the range of 900–200 nm at room temperature. The solution concentrations ranged from 0.02 to 2.0 mM, and quartz cells (path length: 1.0 cm) were used. Electron paramagnetic resonance (EPR) spectra were recorded on a Bruker EMX-nano X-band EPR spectrometer at 77 K using 1 mM solutions of CuSV-Imi, CuSS-Imi, CuSV@lysozyme, and CuSS@lysozyme in quartz tubes as samples in quartz tubes. The cyclic voltammograms were measured by ALS/DY2323 BI-POTENTIOSTAT in a 0.1 M phosphate buffer solution (pH 7.0). Glassy carbon, Pt wire, and Ag/AgCl were used as the working, counter, and reference electrodes, respectively. The CV curves of CuSV-Imi (1.0 mM), CuSS-Imi (1.0 mM), CuSV@Lysozyme (1.0 mM CuSV +1.0 mM lysozyme), and CuSS@lysozyme (1.0 mM CuSS +1.0 mM lysozyme) were measured over four cycles under a nitrogen atmosphere at 298 K with a sweep rate of 100 mV/s. Fluorescence spectra were measured using a Jasco FP-6200 spectrofluorometer. The fluorescence wavelengths were in the range of 220–660 nm. The excitation wavelength was 260 nm. The concentrations of the solutions were in the range of 0–4.0 μM (CuSV, CuSS) + 0 or 4.0 μM (lysozyme).

### 2.4 X-ray crystallographic analysis

Single crystals of CuSV and CuSV-Imi were glued on top of the glass fibers and coated with a thin layer of epoxy resin to obtain the diffraction data. The intensity data were collected on a Bruker APEX2 CCD diffractometer with graphite-monochromated Mo Kα radiation (λ = 0.71073 Å). Data analysis was performed using the SAINT software package. The structures were solved by direct methods using SHELXS-97, expanded by Fourier techniques, and refined by full-matrix least-squares methods based on F^2^ using SHELXL-97 (Sheldrick, 2008). The empirical absorption correction was applied using the SADABS program. All non-hydrogen atoms were readily located and refined using anisotropic thermal parameters. All hydrogen atoms were located in geometrically calculated positions and refined using riding models.

### 2.5 Evaluation of SOD activities

The SOD activities of the Cu^2+^ complexes CuSV-Imi, CuSS-Imi, CuSV@lysozyme, and CuSS@lysozyme were evaluated using the WST SOD assay kit (Dojindo, Tokyo, Japan), which is based on the xanthine-xanthine oxidase method, according to the manufacturer’s instructions. The evaluation was performed in phosphate buffer (0.1 M, pH = 7.0) at 310 K with 2-(4-Iodophenyl)-3-(4-nitrophenyl)-5-(2,4-disulfophenyl)-2H-tetrazolium monosodium salt as indicator. IC_50_ values were determined by inhibition rates of 2000, 400, 80 and 16 μM solutions of the Cu^2+^ complexes. Absorbance was measured at 450 nm using a JASCO V-570 UV-vis spectrophotometer.

### 2.6 Computational details

All calculations were carried out with the Gaussian 09 package (Frisch et al., 2009) and all geometries of O_2_^−^-bound Cu^2+^ complexes in the triplet (S = 1) state were optimized at the density functional theory (DFT) level using B3LYP (Lee et al., 1988) functionals with 6-31+G(d) basis set (Clark et al., 1983) for all atoms. GaussView 5 (Dennington et al., 2009) was used to generate the starting structures and to visualize the optimized structures.

## 3 Results and discussion

### 3.1 Syntheses and crystal structures of the Cu^2+^ complexes

The ligands of the Cu^2+^ complexes were prepared by a reaction between salicylaldehyde and the corresponding *L*-amino acids (*L*-valine and *L*-serine). An equivalent amount of copper (II) acetate was added to the ligand solutions to form green CuSV and CuSS crystals. From the two Cu^2+^ complexes, a single CuSV crystal suitable for X-ray crystallographic analysis was obtained.

**FIGURE 3:**
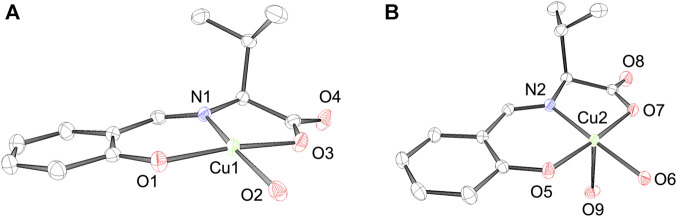
ORTEP drawings of Cu^2+^ complex CuSV with a **(A)** 4-coordinated and a **(B)** 5-coordinated structure. The thermal ellipsoids are drawn at 50% probability. Hydrogen atoms are omitted for clarity. Selected bond length (Å) and bond angles (degree): Cu1−N1 = 1.9205 (16), Cu1−O1 = 1.8955 (15), Cu1−O2 = 1.9339 (16), Cu1−O3 = 1.9629 (15), N1−Cu1−O1 = 94.40 (7), O1−Cu1−O2 = 94.04 (7), O2−Cu1−O3 = 88.01 (7), N1−Cu1−O3 = 82.90 (7), N1−Cu1−O2 = 170.16 (7), O1−Cu1−O3 = 171.32 (7), for structure **(A)**, Cu2−N2 = 1.9243 (17), Cu2−O5 = 1.9432 (14), Cu2−O6 = 1.9411 (15), Cu2−O7 = 1.9956 (14), Cu2−O9 = 2.3663 (16), N2−Cu2−O5 = 92.86 (6), O5−Cu2−O6 = 89.41 (7), O6−Cu2−O7 = 95.00 (7), N2−Cu2−O7 = 82.74 (6), N2−Cu2−O9 = 93.05 (7), O5−Cu2−O9 = 97.98 (6), O6−Cu2−O9 = 86.56 (7), O7−Cu2−O9 = 82.34 (6), N2−Cu2−O6 = 177.73 (7), O5−Cu2−O7 = 175.60 (6), for structure **(B)**, respectively.

According to X-ray analysis, the crystal system of CuSV was orthorhombic, and judging from the systematic absence, the space group was *P2*_*1*_ *2*_*1*_ *2*_*1*_ (Akiyama et al., 2023). In the unit cell, we observed two Cu^2+^ complexes with a four- and five-coordinated structure, as shown in Figure 3. CuSV with a four-coordinated structure was bound by the phenolato O atom, carboxylato O atom, and imino N atom of the ligand and one water molecule on the equatorial plane. Based on the sum of the bond angles around the Cu^2+^ center (359.4°), the four-coordinated unit was concluded to have a square planar structure with an extremely small distortion. In contrast, the other Cu^2+^ complex with a five-coordinated structure was additionally coordinated with another water molecule at the apical position of the plane. Although the coordination of the apical water molecule was too weak (Cu2−O9 = 2.3663 (16), the Cu−N and Cu−O bonds were slightly longer than those of the four-coordination unit. The weak coordination of the apical water molecule is consistent to the elemental analysis of the powdered CuSV. According to the elemental analysis, powdered CuSV contains only one water molecule, indicating the apical water molecule is easily leave from the powdered CuSV. The small τ value (τ = 0.04) indicates that the structure of the five-coordinated unit is square pyramidal (Addison et al., 1984). These crystal structures are similar with previously reported Cu^2+^ complexes with the similar amino acid moiety (Katsuumi et al., 2020; Otani et al., 2021; Suzuki et al., 2023).

**FIGURE 4:**
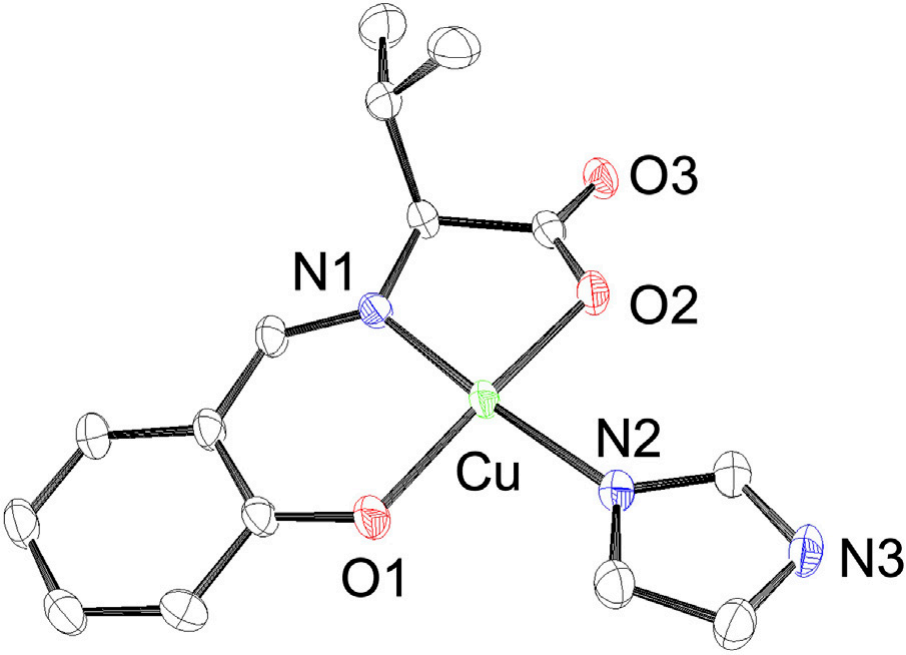
ORTEP drawings of Cu^2+^ complex CuSV-Imi with thermal ellipsoids drawn at 50% probability. Hydrogen atoms are omitted for clarity. Selected bond length (Å) and bond angles (degree): Cu−N1 = 1.927 (3), Cu−O1 = 1.909 (3), Cu−N2 = 1.961 (3), Cu−O2 = 1.971 (3), N1−Cu−O1 = 94.33 (12), O1−Cu−N2 = 91.54 (12), N2−Cu−O2 = 90.53 (11), N1−Cu−O2 = 82.91 (11), N1−Cu−N2 = 172.24 (13), O1−Cu−O2 = 171.11 (11).

Because we estimated the His15 side chain of lysozyme to be the Cu^2+^ complex-binding site, we also prepared imidazole-binding CuSV and CuSS (CuSV-Imi and CuSS-Imi) as lysozyme-bound structural models. CuSV-Imi and CuSS-Imi were synthesized by the reaction between CuSV/CuSS and an equivalent amount of imidazole. The reaction solutions were kept at 298 K, and dark green crystals were observed. Of the two crystals, CuSV-Imi yielded single crystals suitable for crystallographic analysis. Based on crystallographic analysis and the systematic absence, the crystal system and space group of CuSV-Imi were monoclinic and *P2*_*1*_, respectively. The crystal structure of CuSV-Imi is shown in Figure 4. Upon reaction with imidazole, the water molecules of CuSV on the equatorial plane are replaced to form a square planar structure. Based on the bond angles around the Cu^2+^ center (359.3°), the square planar structure exhibits a small distortion. Through the coordination of imidazole on the plane, the other Cu−N and Cu−O bonds were slightly stretched. These bond elongations can be explained by the stronger electron donation of the imidazole compared to that of the water molecule. These structural features are similar to those of the previously reported Cu^2+^ complexes (Watanabe and Akitsu, 2012; Takeshita et al., 2015; Furuya et al., 2023), which has the same framework as CuSV-Imi.

### 3.2 Spectroscopic properties of the Cu^2+^ complexes

CuSV showed *ν* (C=N) vibration at 1,639 cm^−1^ and symmetric and asymmetric *ν* (COO^−^) vibrations at 1,468 cm^−1^ and 1,600 cm^−1^, respectively. The significant difference between symmetric and asymmetric ν(COO^−^) vibrations (≈140 cm^−1^) reflects the monodentate coordination of the carboxyl group (Reddy et al., 2011). CuSV also showed *ν* (O−H) vibration at 3,232 cm^−1^, which was due to the coordinating water molecule on the equatorial plane. In contrast, due to the replacement of water molecules and an imidazole on the equatorial plane, CuSV-Imi showed *ν* (C=N), symmetric and asymmetric *ν* (COO^−^) vibrations in slightly lower energy regions (1,632 cm^−1^, 1,465 cm^−1^, and 1,586 cm^−1^ respectively), while the *ν* (O−H) vibration was disappeared. The disappearance of the *ν* (O−H) vibration indicates that no water molecule is contained in the crystal of CuSV-Imi, which is consistent to the elemental analysis of CuSV. CuSS also showed *ν* (C=N), symmetric/asymmetric *ν* (COO^−^) vibrations at 1,628 cm^−1^, 1,465 cm^−1^, and 1,602 cm^−1^, respectively. In addition, CuSS showed *ν* (O−H) vibrations at 3,132 cm^−1^, 3,236 cm^−1^, and 3,320 cm^−1^. These *ν* (O−H) vibrations were assigned to hydroxyl groups of serine side chain, and coordinating/noncoordinating water molecules. Upon replacement of water molecule on the equatorial plane to imidazole, CuSS-Imi also showed *ν* (C=N), symmetric and asymmetric *ν* (COO^−^) vibrations in slightly lower energy regions (1,626 cm^−1^, 1,462 cm^−1^, 1,600 cm^−1^ respectively), while *ν* (O−H) vibrations assigned as coordinating and noncoordinating water molecules (3,236 cm^−1^, and 3,320 cm^−1^) were disappeared. These disappearances of *ν* (O−H) vibrations assigned as the water molecules are also consistent to the elemental analysis of CuSS-Imi.

**FIGURE 5:**
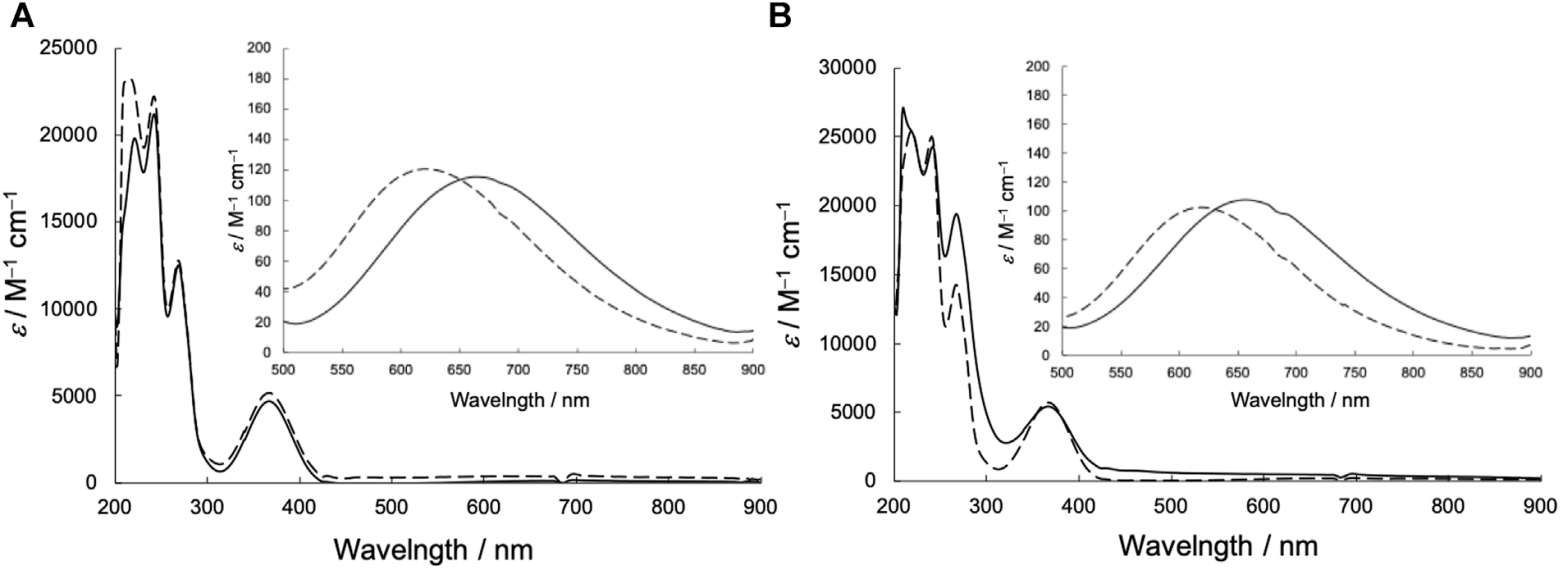
UV-vis spectra in methanol solution (0.02 mM) of **(A)** CuSV (solid line), CuSV-Imi (dashed line) and **(B)** CuSS (solid line), CuSS-Imi (dashed line). Insert: Expanded UV-vis spectra in 500–800 nm region (2 mM).

The UV-vis spectra of the Cu^2+^ complexes are shown in Figure 5. UV-vis spectroscopic measurements revealed that CuSV showed absorption maxima at 266 nm (*ε* = 12,000 M^−1^ cm^−1^), 368 nm (*ε* = 5600 M^−1^ cm^−1^), and 664 nm (*ε* = 140 M^−1^ cm^−1^) assigned to π−π*, n−π*, and d-d transitions, whereas CuSS showed absorption maxima at 270 nm (*ε* = 19,000 M^−1^ cm^−1^), 367 nm (*ε* = 5400 M^−1^ cm^−1^), and 658 nm (*ε* = 110 M^−1^ cm^−1^). The similar UV-vis spectral features of CuSV and CuSS indicate that both Cu^2+^ complexes have similar structures in solution. In contrast, CuSV-Imi exhibited π−π*, n−π*, and d-d transitions at 269 nm (*ε* = 13,000 M^−1^ cm^−1^), 374 nm (*ε* = 4900 M^−1^ cm^−1^), and 622 nm (*ε* = 120 M^−1^ cm^−1^), whereas CuSS-Imi exhibited them at 269 nm (*ε* = 14,000 M^−1^ cm^−1^), 367 nm (*ε* = 5700 M^−1^ cm^−1^), 610 nm (*ε* = 130 M^−1^ cm^−1^), respectively. Upon replacing the water molecule on the equatorial plane with imidazole, both CuSV-Imi and CuSS-Imi exhibited d-d transitions in a higher energy region (622 and 610 nm) than CuSV and CuSS (664 and 658 nm). The higher energy shifts of the d-d transitions can be explained by the strong coordination of imidazole with the Cu^2+^ center. Higher energy shifts of their d-d transitions upon the replacement of water molecules with imidazole have been reported theoretically and experimentally (Furuya et al., 2023).

**FIGURE 6:**
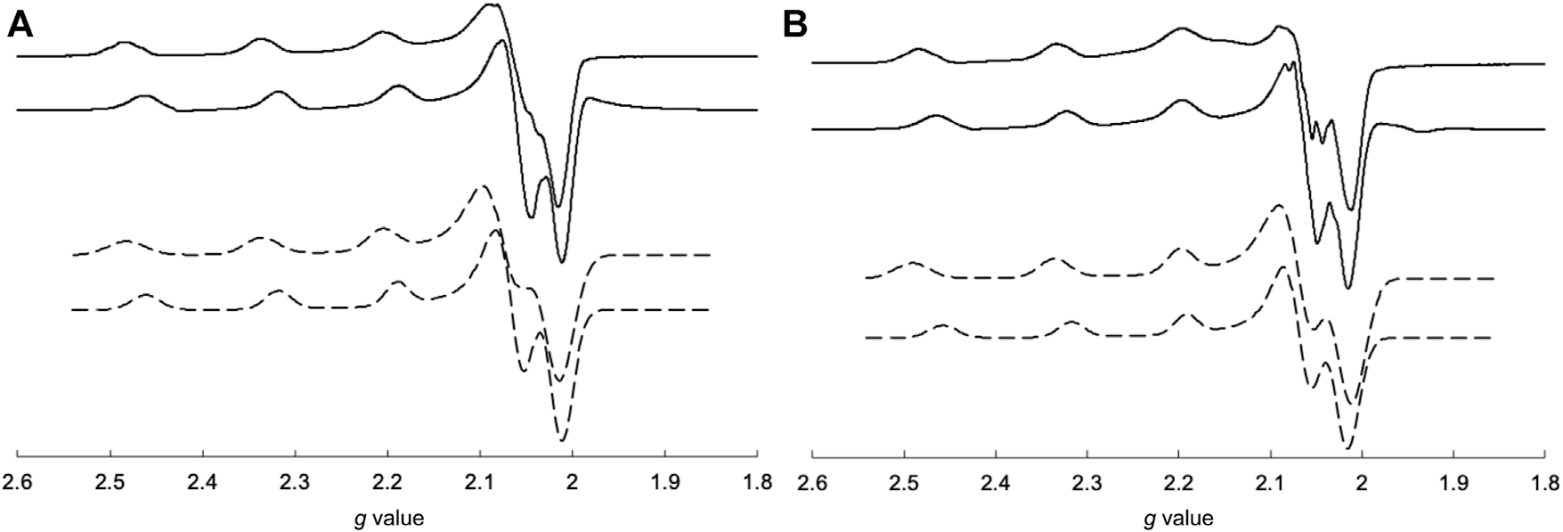
Comparisons of measured (solid lines) and simulated (dashed lines) EPR spectra of **(A)** CuSV (top), CuSV-Imi (bottom) and **(B)** CuSS (top), CuSS-Imi (bottom). All samples were prepared as 1 mM solutions in 0.1 M phosphate buffer (pH: 7.0) in quarts tubes and measured at 77 K.

The EPR spectra of the Cu^2+^ complexes are shown in Figure 6. CuSV had g_//_ and g_⊥_ values of 2.269 (|A_//_| = 173 G) and 2.065 (|A_⊥_| ≈ 20 G), respectively, whereas the g_//_ and g_⊥_ values of CuSS were 2.265 (|A_//_| = 183 G) and 2.067 (|A_⊥_| ≈ 10 G), respectively. Both Cu^2+^ complexes show larger g_//_ values than their g_⊥_ values, indicating that the Cu^2+^ centers have unpaired electrons in their dx^2^-y^2^ orbitals. These features are characteristic of Cu^2+^ complexes with square planar or pyramidal structures (Ramakrishnan et al., 2009). On the other hand, CuSV-Imi and CuSS-Imi showed g_//_ and g_⊥_ values of 2.252 (|A_//_| = 173 G) and 2.063 (|A_⊥_| ≈ 10 G), and 2.253 (|A_//_| = 169 G) and 2.065 (|A_⊥_| ≈ 10 G), respectively. When the water molecule at the equatorial position was replaced with imidazole, both Cu^2+^ complexes exhibited smaller g_//_ values. These smaller g_//_ shifts can be explained by the strong coordination of imidazole at the equatorial position.

### 3.3 Formation of hybrid proteins composed of the Cu^2+^ complexes and lysozyme

**FIGURE 7:**
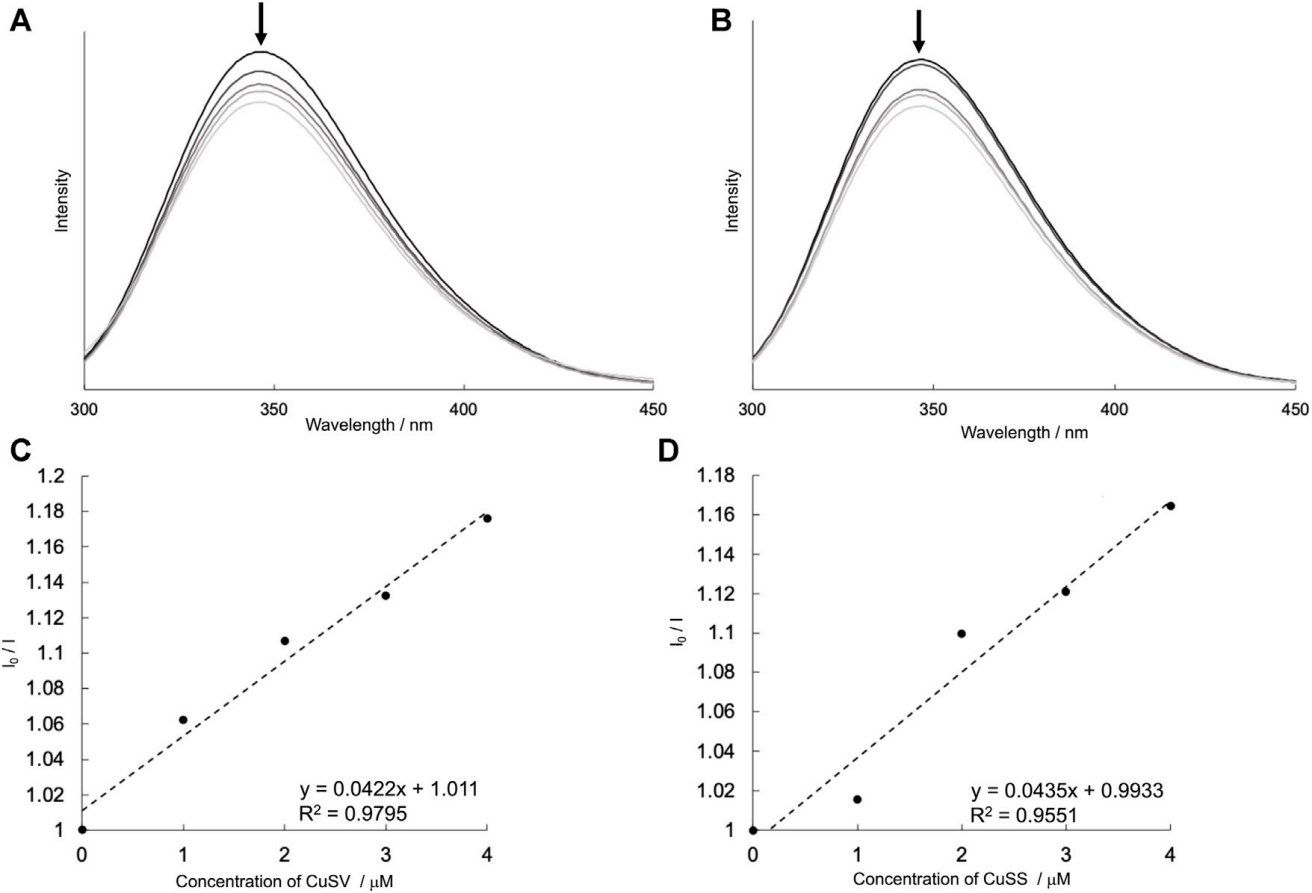
Emission spectra (λ_ex_ = 260 nm) of lysozyme (4 μM) in presence of various concentrations of **(A)** CuSV (0–4 μM), **(B)** CuSS (0–4 μM), and their Stern–Volmer plots [**(C,D)**, respectively]. These I_0_/I values were plotted by using their emission maxima (λ_em_ = 346 nm). All samples were prepared as 0.1 M phosphate buffer solution (pH = 7.0) and measured at 298 K.

To confirm the formation of the hybrid proteins composed of Cu^2+^ complexes and lysozyme, the emission spectra of lysozyme solutions containing various concentrations of CuSV and CuSS were measured. As shown in Figures 7A, B, the lysozyme solutions produced emissions at 346 nm. These lysozyme solutions produced weaker emissions when the Cu^2+^ complex, CuSV or CuSS, was present at higher concentrations. In addition, based on the positive slopes obtained from the Stern–Volmer plots (Figures 7C, D), interactions and energy transitions between these Cu^2+^ complexes and lysozyme were suggested.

**FIGURE 8:**
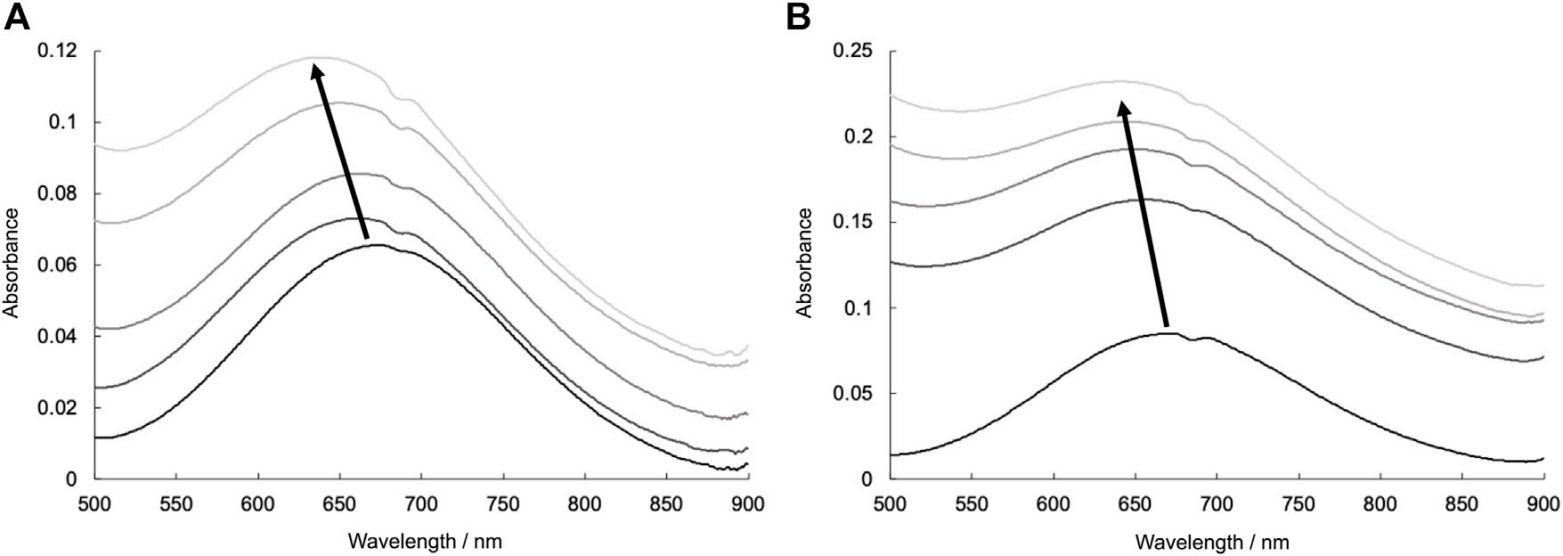
UV-vis spectra due to d-d transitions of **(A)** CuSV (0.8 mM) and **(B)** CuSS (0.8 mM) in presence of various concentrations of lysozyme (0–0.8 mM). All samples were prepared as 0.1 M phosphate buffer solution (pH = 7.0) and measured at 298 K.

UV-vis spectral investigations also indicated the coordination of lysozyme with Cu^2+^ complexes. The UV-vis spectra of CuSV and CuSS in the presence of various concentrations of lysozyme are shown in Figure 8. CuSV and CuSS exhibited their d-d transitions at 676 and 670 nm, respectively, in phosphate buffer. These absorptions shifted to a higher-energy region when lysozyme was present. These higher-energy shifts were also obtained by replacing water molecules on the equatorial planes of CuSV and CuSS with imidazole (Figure 5). Judging from these UV-vis spectral behaviors similar to those of previously reported CuST-bound lysozyme whose crystal structure are revealed (Furuya et al., 2023), coordination of the His15 side chain to the equatorial position of the Cu^2+^ centers of CuSV and CuSS are suggested.

**FIGURE 9:**
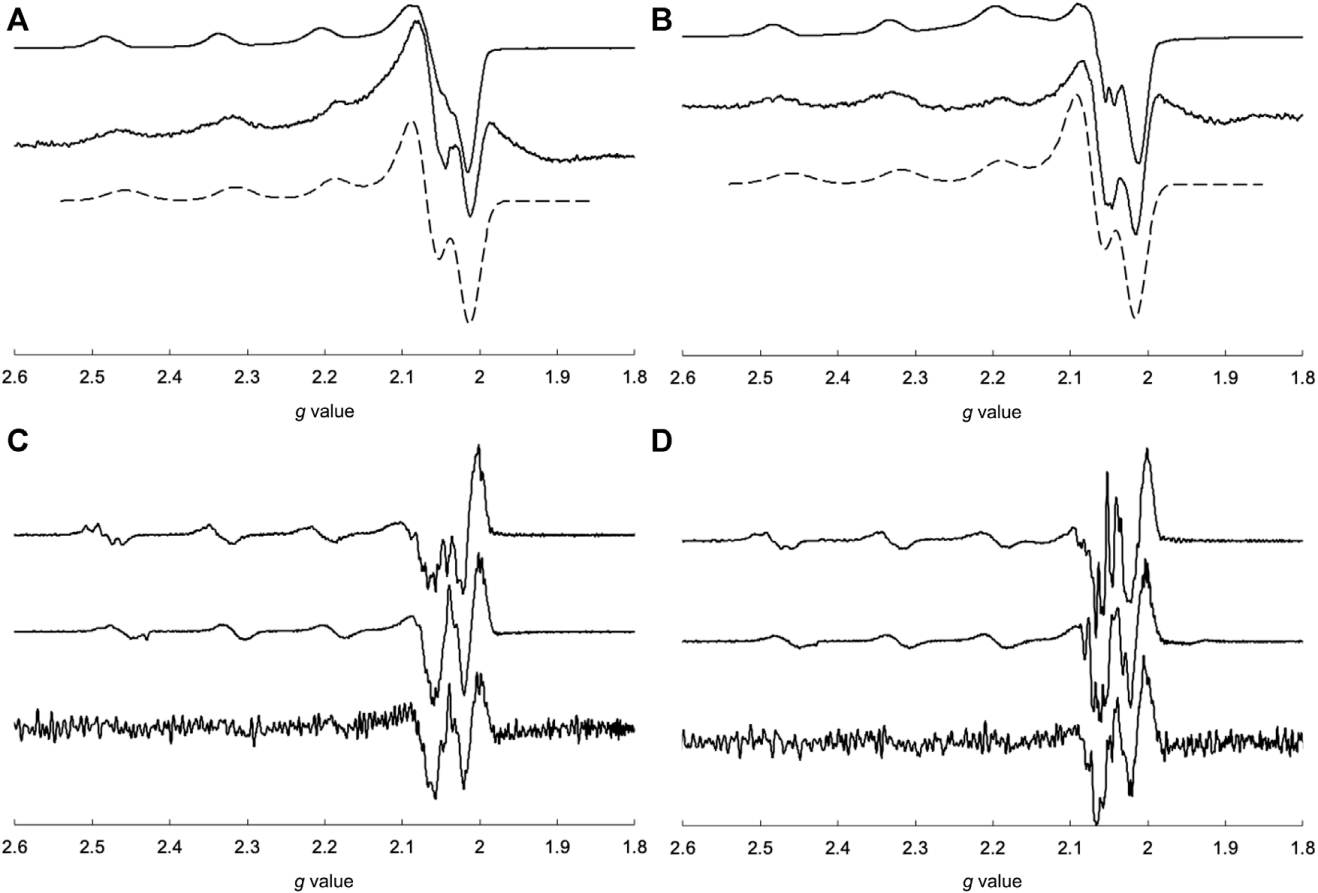
EPR spectra of **(A)** CuSV (top), measured and simulated 1:1 mixture of CuSV and lysozyme (middle, bottom), **(B)** CuSS (top), measured and simulated 1:1 mixture of CuSS and lysozyme (middle, bottom), and differential EPR spectra of **(C)** CuSV (top), CuSV-Imi (middle), 1:1 mixture of CuSV and lysozyme (bottom), **(D)** CuSS (top), CuSS-Imi (middle), 1:1 mixture of CuSS and lysozyme (bottom). All samples were prepared as 0.8 mM solution in 0.1 M phosphate buffer solution (pH = 7.0) in quarts tubes and measured at 77 K.

EPR spectroscopy is useful for predicting the electronic structures around Cu^2+^ centers. The EPR spectral behavior of the Cu^2+^ complex in the presence of lysozyme is shown in Figure 9. As mentioned above, CuSV had g_//_ and g_⊥_ values of 2.269 (|A_//_| = 173 G) and 2.065 (|A_⊥_| ≈ 20 G), respectively, whereas CuSS has values of 2.265 (|A_//_| = 183 G) and 2.067 (|A_⊥_| ≈ 10 G), respectively. They showed smaller g_//_ values (2.251 (|A_//_| = 171 G) for CuSV and 2.255 (|A_//_| = 170 G) for CuSS) when the equivalent amounts of lysozyme were presented, while their g_⊥_ values showed only slight changes (2.065 (|A_⊥_| ≈ 10 G) for CuSV and 2.066 (|A_⊥_| ≈ 10 G) for CuSS). As mentioned, smaller shifts in the g_//_ values were also observed for the imidazole coordination to CuSV and CuSS on the equatorial plane (Figure 6). Differential EPR spectra of CuSV and CuSS showed hyperfine splitting due to coordination of imino N atom of the ligands in their g_⊥_ regions. CuSV-Imi and CuSS-Imi also showed hyperfine splitting in their g_⊥_ regions with different splitting pattern from those of CuSV and CuSS, attributed to the coordination of imidazole on the equatorial plane. Furthermore, both CuSV and CuSS showed similar differential EPR spectra to CuSV-Imi and CuSS-Imi when an equivalent of lysozyme was presented. These EPR spectroscopic findings also indicated that CuSV and CuSS were bound to lysozyme by the coordination of the imidazole group of His15 on their equatorial plane.

**FIGURE 10:**
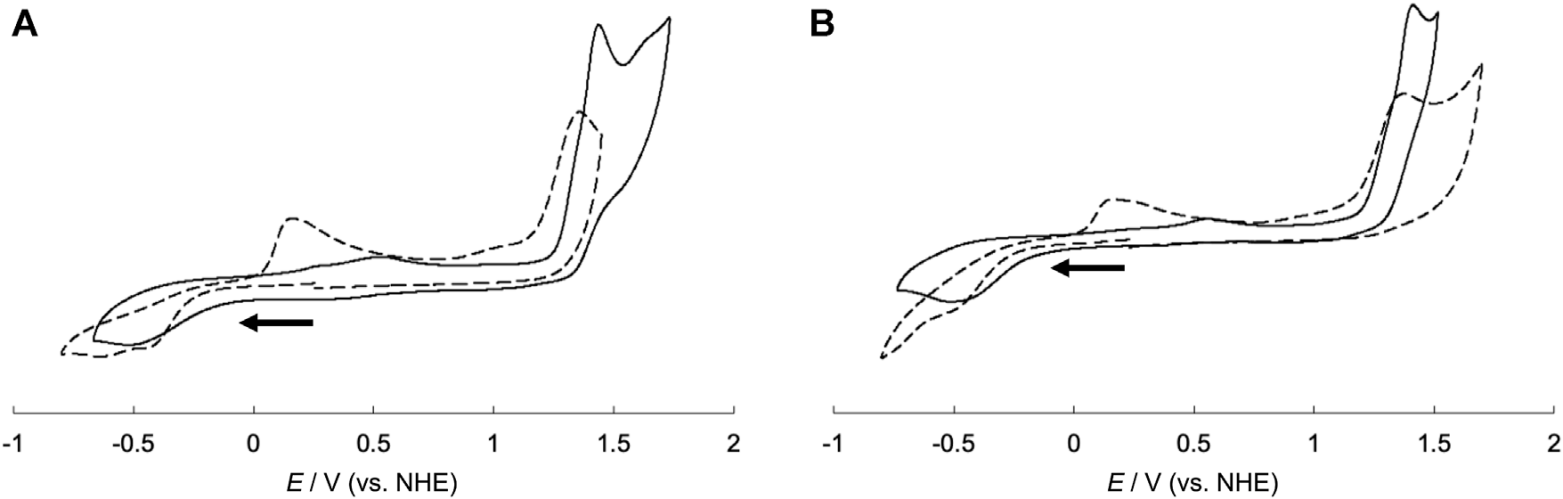
Cyclic voltammograms of **(A)** CuSV-Imi (solid line), CuSV@lysozyme (dashed line) and **(B)** CuSS-Imi (solid line), CuSS@lysozyme (dashed line). All samples were prepared as 1.0 mM solution in 0.1 M phosphate buffer solution (pH = 7.0). Glassy carbon, Pt wire, and Ag/AgCl electrodes were used as the working, counter, and reference electrodes, respectively and measured with a sweep rate of 100 mV/s. Potentials were converted from Ag/AgCl to NHE.

To investigate the electrochemical properties of the hybrid proteins, their cyclic voltammograms were measured and compared with those of CuSV-Imi and CuSS-Imi. A comparison of the voltammograms is presented in Figure 10. CuSV-Imi exhibits an irreversible redox pair. The reduction and oxidation waves were found at −0.51 V and +0.56 V (*ΔE* = 1.07 V), respectively. Based on the rest potential (+0.51 V) and the initial scan polarity (negative), the redox pair was identified as a Cu^2+^/Cu^+^. The significant peak separation (*ΔE* = 1.07 V) indicates a slow electron transfer caused by structural changes around the metal center during the redox process. CuSS-Imi also showed the reduction and oxidation waves due to the Cu^2+^/Cu^+^ process at −0.47 V and +0.57 V (*ΔE* = 1.04 V), respectively. Based on the comparison of the redox potentials and their separation, the hydroxyl group of CuSS-Imi has only a small effect on its electrochemical properties. On the other hand, the CuSV-bound lysozyme (CuSV@lysozyme) had Cu^2+^/Cu^+^ reduction and oxidation waves at −0.43 V and +0.16 V.

From the formation of the adduct, CuSV@lysozyme produced a smaller peak separation (*ΔE* = 0.59 V) than CuSV-Imi (*ΔE* = 1.07 V). CuSS-bound lysozyme (CuSS@lysozyme) also produced reduction and oxidation waves due to the Cu^2+^/Cu^+^ process at −0.48 V and +0.17 V and a smaller peak separation (*ΔE* = 0.65 V) than CuSS-Imi. These smaller peak separations suggest that the structural rearrangement around the metal center upon Cu^2+^/Cu^+^ processes were decreased by lysozyme hybridization. Our group previously reported the crystal structure of a lysozyme-bound Cu^2+^ complex (CuST@lysozyme) (Furuya et al., 2023). Crystallographic analysis revealed that the Cu^2+^ complex, CuST, was coordinated by the imidazole group of the His15 side chain. In addition, the Cu^2+^ center was fixed by weak coordination of the Thr89 side chain. Owing to the weak coordination, the Cu^2+^ centers of lysozyme-bound CuSV and CuSS can be fixed and decrease structural changes in the Cu^2+^/Cu^+^ process. The diminution of structural changes around the metal centers during the Cu^2+^/Cu^+^ process can favor the disproportionation activity of the O_2_^−^ anion.

### 3.4 SOD activity evaluations of the hybrid proteins composed of the Cu^2+^ complexes and lysozyme

Spectroscopic measurements suggested that both CuSV and CuSS formed hybrid proteins with lysozymes. Therefore, we investigated the SOD activity of these hybrid proteins (CuSV@lysozyme and CuSS@lysozyme) to evaluate the effects of hybridization with lysozymes. SOD activity was evaluated as IC_50_ values obtained using the xanthine/xanthine oxidase method. The results of the SOD activity evaluation are summarized in Table 1. First, we compared the SOD activities of CuSV-Imi and CuSS-Imi. CuSS-Imi showed higher SOD activity (IC_50_ = 684 μM) than CuSV-Imi (IC_50_ = 1,203 μM). Based on the comparison of their structures, the SOD activity of CuSS-Imi can be affected by the hydroxyl group on the side chain of the serine moiety. In contrast, through hybridization with lysozyme, CuSV@lysozyme and CuSS@lysozyme had higher SOD activities (IC_50_ = 845 μM and 326 μM, respectively) than CuSV-Imi and CuSS-Imi. Based on the poor SOD activity of lysozyme (IC_50_ >> 2000 μM), the improvements in SOD activity were due to the cooperative effects of the Cu^2+^ complexes and lysozyme.

**TABLE 1:** SOD activities of the Cu^2+^ complexes and hybrid proteins.

Sample	IC_50_/μm
CuSv-Imi	1,203
CuSS-Imi	684
CuSV@Iysozyme	845
CuSS©lysozyme	326
Lysozyme	>>2,000
CuZnSOD	<16

### 3.5 Theoretical investigations

**FIGURE 11:**
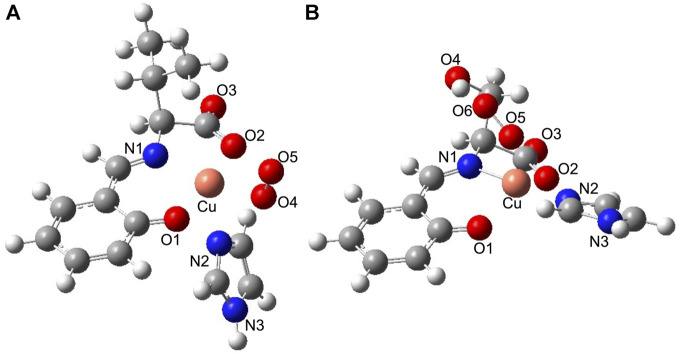
Theoretically proposed structures of **(A)** O_2_^−^-bound CuSV-Imi and **(B)** O_2_^−^-bound CuSS-Imi. Selected bond length (Å) and bond angles (degree): Cu−N1 = 2.022, Cu−O1 = 2.038, Cu−N2 = 2.397, Cu−O2 = 2.048, Cu−O4 = 1.975, N1−Cu−O1 = 90.156, O1−Cu−O4 = 92.369, O4−Cu−O2 = 96.667, O2−Cu−N1 = 81.359, N1−Cu−N2 = 106.462, O1−Cu−N2 = 90.077, O4−Cu−N2 = 88.433, O2−Cu−N2 = 89.232, N1−Cu−O4 = 164.894, O1−Cu−O2 = 170.915, for structure **(A)**, and Cu−N1 = 1.940, Cu−O1 = 2.114, Cu−N2 = 1.993, Cu−O2 = 2.113, Cu−O5 = 2.169, N1−Cu−O1 = 91.113, O1−Cu−N2 = 93.168, N2−Cu−O2 = 92.353, O2−Cu−N1 = 80.806, N1−Cu−O5 = 98.791, O1−Cu−O5 = 95.293, N2−Cu−O5 = 86.225, O2−Cu−O5 = 128.491, N1−Cu−N2 = 173.086, O1−Cu−O2 = 136.136, for structure **(B)**, respectively.

According to comparisons of the SOD activities of the Cu^2+^ complexes and their lysozyme adducts, the presence of a hydroxyl group on the Cu^2+^ complex and hybridization with lysozyme improved SOD activity. To understand their SOD activity, theoretically optimized structures of O_2_^−^-bound Cu^2+^ complexes and their lysozyme adducts were compared. The optimized structures of O_2_^−^-bound CuSV-Imi and CuSS-Imi are shown in Figure 11. To the Cu^2+^ center of CuSV-Imi, a O_2_^−^ anion coordinated on the equatorial position to form square pyramidal structure (τ = 0.10). By the coordination of O_2_^−^ anion, the imidazole molecule was kicked out to the axial position and weakly coordinated (2.397 Å) to the Cu^2+^ center. The drastic structural change can prevent fast electron transfer between the metal center and superoxide. On the other hand, the Cu^2+^ center of CuSS-Imi was coordinated by a O_2_^−^ anion to form distorted trigonal bipyramidal structure (τ = 0.62). Although the structure of the Cu^2+^ center was distorted by the coordination of a O_2_^−^ anion, the imidazole molecule on the equatorial position of CuSS-Imi was not kicked out. In addition, the coordinated O_2_^−^ anion formed hydrogen bond with hydroxyl group of the side chain of the serine moiety. Attributed to these structural features, the O_2_^−^-bound CuSS-Imi showed 128.9 kJ/mol lower energy than that of O_2_^−^-bound CuSV-Imi. These theoretical findings consistent to the higher SOD activity of CuSS-Imi than that of CuSV-Imi.

### 3.6 Discussion on the higher SOD activity of CuSS@lysozyme

**FIGURE 12:**
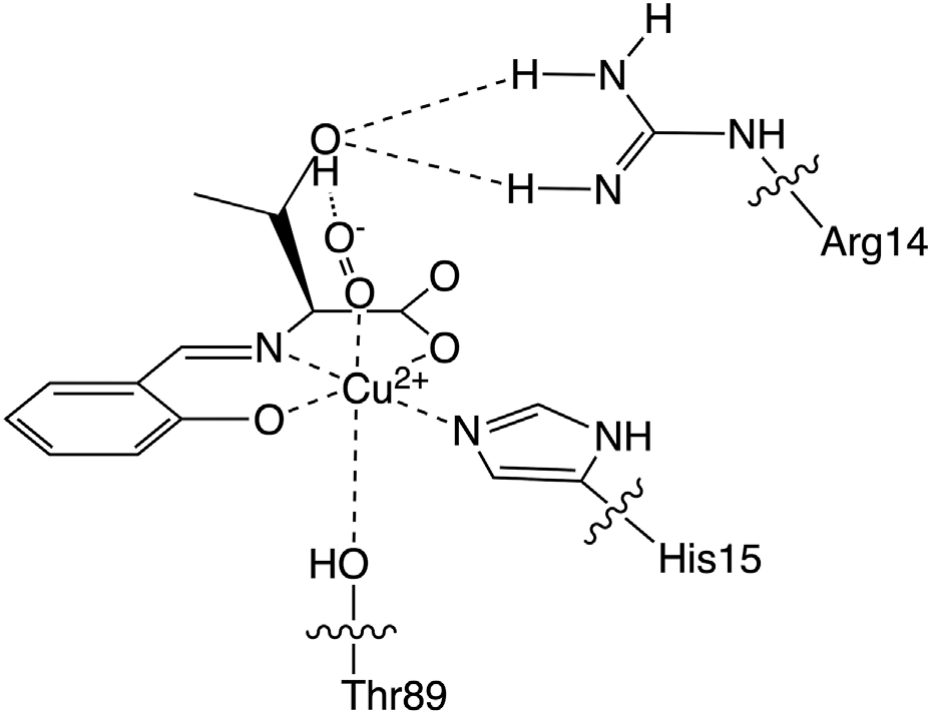
Estimated structure of O_2_^−^-bound CuSS@lysozyme.

Based on these experimental and theoretical investigations, the higher SOD activity of CuSV@lysozyme than that of CuSS@lysozyme can be explained as follows: i) Judging from the previously reported crystal structure of CuST@lysozyme (Furuya et al., 2023) and spectroscopic behaviors of CuSV and CuSS upon presence of lysozyme, the imidazole group of His15 residue and hydroxyl group of Thr89 bind to the Cu^2+^ center of CuSS on the equatorial and axial position, respectively. The theoretical investigation suggested that hydrogen bonds can be formed between the O_2_^−^ anion and the hydroxyl group owing to the serine moiety of CuSS when the O_2_^−^ anion coordinates with the metal center, as shown in Figure 12. The theoretical investigation also suggested that the coordination of the O_2_^−^ anion to the metal center can be stabilized and promoted through the hydrogen bond. ii) By forming a hybrid protein with lysozyme, in addition to the coordination of the imidazole group of the His15 side chain with the metal center at the equatorial position, the hydroxyl group of Thr89 is weakly coordinated at the axial position. Electrochemical behaviors of CuSV@lysozyme and CuSS@lysozyme implied that the weakly coordinated Thr89 residue can contribute to reduce the structural changes during the Cu^2+^/Cu^+^ process. The reduced structural changes during the Cu^2+^/Cu^+^ process can improve electron transfer between O_2_^−^ anions and enhance the SOD activity of the metal center.

## 4 Conclusion

In this study, we prepared Cu^2+^ complexes, CuSV and CuSS, in the absence/presence of hydroxyl groups, and their imidazole-bound compounds, CuSV-Imi and CuSS-Imi, to reveal the effects of hydroxyl groups on SOD activity. Crystallographic analysis and spectroscopic measurements suggested that the imidazole group coordinated with the Cu^2+^ ions at the equatorial positions of CuSV and CuSS, both in the solid state and in solution. Based on these properties and spectroscopic behaviors in the presence of lysozyme on CuSV and CuSS, the imidazole group of His15 in lysozyme can coordinate with the Cu^2+^ center in its equatorial position to form hybrid proteins with lysozyme. Electrochemical measurements indicated that the structural changes around the Cu^2+^ center of CuSV and CuSS during the Cu^2+^/Cu^+^ process were decreased by the formation of hybrid proteins with lysozyme.

A comparison of the SOD activities of CuSV-Imi and CuSS-Imi indicated that the hydroxyl group of CuSS-Imi played an important role in the disproportionation of the O_2_^−^ ion. In addition, hybridization of the Cu^2+^ complexes CuSV and CuSS with lysozyme resulted in higher SOD activity than that of CuSV-Imi and CuSS-Imi. These improvements in SOD activity suggest that there are cooperative effects between Cu^2+^ complexes and lysozyme. These improvements in SOD activity can be explained by the stabilization of the O_2_^−^-bound metal center caused by the hydrogen bond between the coordinating O_2_^−^ ion and the hydroxyl group. The diminution of the structural changes upon the redox of the metal center caused by the weak coordination of the hydroxyl group of the Thr89 side chain with the metal center also contributes to the improvement of the SOD activity.

## Data Availability

Publicly available datasets were analyzed in this study. This data can be found here: CCDC code of CuSV-Imi (2310763).
